# Enhancing Pathology Knowledge for Biomedical Scientists: Pilot Cohort Study of a Comprehensive Self-Paced Tutorial for Bridging Fundamental Concepts With Translational Applications

**DOI:** 10.2196/84903

**Published:** 2026-05-21

**Authors:** Jennifer Sells, Leticia C Clemente, David P Farris, Mary L Sizemore, Ignacio I Wistuba, Rama Soundararajan, Maria G Raso

**Affiliations:** 1 Department of Education, Development & Innovation, The University of Texas MD Anderson Cancer Center, Houston, TX, United States; 2 Department of Translational Molecular Pathology, The University of Texas MD Anderson Cancer Center, 2130 W Holcombe Blvd., Houston, TX, 77030, United States, 1 281-797-9234; 3 Interdisciplinary Translational Education and Research Training (ITERT) Core, The University of Texas MD Anderson Cancer Center, Houston, Texas, United States; 4 Research Medical Library, The University of Texas MD Anderson Cancer Center, Houston, TX, United States

**Keywords:** pathology education, continuing professional development, online learning, biomedical research training, translational medicine

## Abstract

**Background:**

Knowledge of pathology is integral to numerous health care and research disciplines, besides routine laboratory-medicine operations. Despite its importance, there remains a significant gap in structured educational programs designed to provide a fundamental understanding of the pathologic basis of disease for an interdisciplinary body of research and health care professionals. This lack of generalized pathology training may contribute to a broader knowledge deficit in its translational applications.

**Objective:**

To address this unmet educational need, we developed a distinctive pathology training course tailored to cater to a broad array of scientists in basic and translational science.

**Methods:**

Overall, 29 participants from the University of Texas MD Anderson Cancer Center and the Texas Medical Center completed the “Fundamental Pathology for Basic Scientists: A Self-Paced Online Certificate Course” pilot. The course, comprising 24 instructional modules, was delivered over the span of 6 months in an online, asynchronous, and readily-accessible CANVAS learning management format. The educational objectives for each module were organized around organ- and organ-system-specific content, aiming to (1) incorporate the pathological basis of diseases, (2) distinguish clinicopathologic correlations, and (3) recognize the impact of molecular- and translational pathology and associated state-of-the-art diagnostic technology. Participants were voluntarily surveyed both before and after the course to assess its effectiveness.

**Results:**

The preentry survey revealed that participants represented 16 academic departments (with widely varied training backgrounds, career stages, and job descriptions within the biomedical research enterprise), with 76% (22/29) reporting no prior basic pathology training. The exit survey highlighted positive feedback on the course structure, instructors, delivery method, and lecture content. Overall, 100% (29/29) of learners completed the objective knowledge checks for each of the 24 modules. At least 89% (26/29) of learners concurred that each of the 24 modules had delivered its learning objectives. A total of 94% (27/29) of learners concurred that the course enriched their understanding of basic pathology. Additionally, 79% (23/29) of participants reported applying or being able to apply what they had learned to their current roles, and 94% (15/16) of postcourse survey respondents confirmed longer-term benefits 1 year after program completion (increased confidence in addressing projects involving pathology).

**Conclusions:**

The initial outcomes of this pilot course suggest that the asynchronous delivery method was well-suited to participants’ workloads, and that the content was valuable for basic- and translational-scientists from varied training backgrounds seeking to deepen their understanding of fundamental pathology in the context of biomedical research. This work establishes a model for scalable, technology-enabled pathology training adaptable to diverse learners within health care research centers.

## Introduction

In biomedical research, it is essential for basic science scholars, graduate students, and postdoctoral fellows to not only master their research-specific disciplines, but also stay updated with advancements in interdisciplinary related fields that impact their mainstream research focus. One such interdisciplinary area is pathology, which plays a critical role in bridging the gap between basic research and its clinical applications. A broad understanding of this field is an indispensable asset for basic scientists and health care professionals alike. Insights into the pathological basis of disease are vital for uncovering mechanisms of disease progression and identifying potential therapeutic targets in the research laboratory. Similarly, professionals in laboratory medicine rely on pathology to diagnose and monitor diseases, interpret lab results, and contribute effectively to patient care. Although the need to build or update knowledge and skills in pathology is eminent for workers in health care research centers, many unfortunately either find themselves distanced from foundational pathology concepts over time or lack exposure to the latest developments in translational pathology and emerging technologies [1-3]. In addition to these challenges, there are a litany of unmet educational needs and preexisting or unsolved issues plaguing training programs in pathology, spanning decades from the 1980s to the present day: (1) limited resources for training research scientists in pathology, balancing emphasis on molecular vs general pathology topics, (2) lack of attraction to pathology as a dedicated career track for postgraduate students, and (3) attrition of trained pathologists (limited availability of qualified instructors) [4-8]. There is also a significant gap in the availability of local pathology courses designed for a broader range of professionals who have not pursued “formal” pathology training or work outside of traditional academic pathways, yet require a solid foundation in this field to enhance their everyday work. However, well-recognized training program scholars highlight that regardless of the job type, 70% of learning that directly impacts job performance occurs *on* the job, suggesting the dire need for locally accessible pathology learning courses catering to the career needs of an expansive learner population in health care research centers [9].

To directly address this educational gap, we engaged in the intentional development and delivery of a self-paced learning course to serve a broad breadth of learners in the health care workplace, ranging from graduate students and postdoctoral fellows in translational research to research assistants, technicians, basic scientists, histotechnologists, laboratory managers, quality assurance specialists, and data specialists, as well as research faculty. The “Fundamental Pathology for Basic Scientists” course was developed as part of the Interdisciplinary Translational Education and Research Training (ITERT) Core at the University of Texas (UT) MD Anderson Cancer Center (MD Anderson), a platform dedicated to advancing the educational and professional development of the next generation of leaders in translational research [10,11], in partnership with the Department of Education Practice & Workforce Advancement.

The curriculum was tailored to provide participants with a comprehensive awareness of traditional pathology concepts (Robbins & Kumar Basic Pathology, Eleventh Edition, the international gold-standard text) while integrating instruction on modern innovations in molecular pathology and methodologies that are pivotal to new-age translational research (instructors sharing relevant pathology research using state-of-the-art methodologies and instrumentation). The main purpose of this course was to offer a structured opportunity for participants to deepen their understanding of pathology in a research context, revisit core pathology principles, acquire updated knowledge in translational pathology, and, importantly, engage with interdisciplinary workforce professionals and network with a constellation of trained pathology mentors and instructors. This study describes the initial training outcomes of this pilot educational course.

## Methods

### Course Objectives

The primary goals for this course were to (1) address the nonavailability of workplace training in fundamental pathology (that is traditionally limited to degree-awarding academic programs) by creating a nondegree certificate course open to learners from different disciplines in biomedical research, (2) provide a structured learning rubric for understanding the pathological basis of disease in an organ-organ system format, (3) design flexible self-paced and asynchronous learning modules to serve full-time employees in the health care research enterprise, and (4) develop a support network (peer- and tiered-mentoring) for nonpathology health care professionals who indirectly interface with the pathology-domain.

### Course Participants

Recruitment targeted researchers, staff scientists, and health professionals from across the Texas Medical Center in Houston, Texas, interested in completing a structured course on fundamental pathology for their professional development. The 29 individuals who completed all modules of the course encompassed 10 different professional roles across 16 different academic department affiliations enrolled in the course. The largest single-career stage represented was among 8 out of 29 participants (28%) being research postdoctoral fellows (Table S1 in Multimedia Appendix 1). Regarding the institutions of affiliation, 26 out of 29 participants (90%) were from MD Anderson, and 3 out of 29 participants (10%) were from UT Health Houston.

A learner analysis was conducted to gain an understanding of the participants’ training backgrounds and learning objectives. This information was captured in a pre-course survey administered before participants began coursework. Following best practices for conducting a learner analysis, 5 essential concepts were adhered to, in accordance with recommendations by Stefaniak [12]:

1. General characteristics
2. Prior pathology knowledge and prerequisite skills of participants
3. Predispositions pertaining to the topic of pathology
4. Attitude and motivation toward training
5. Characteristics of the group as a whole

This early analysis allowed for a comprehensive snapshot of learner demographics in terms of academic career stage, background experience in pathology, and instructional modules needing content tailoring.

### Course Design, Curriculum, and Delivery

**Figure 1.**
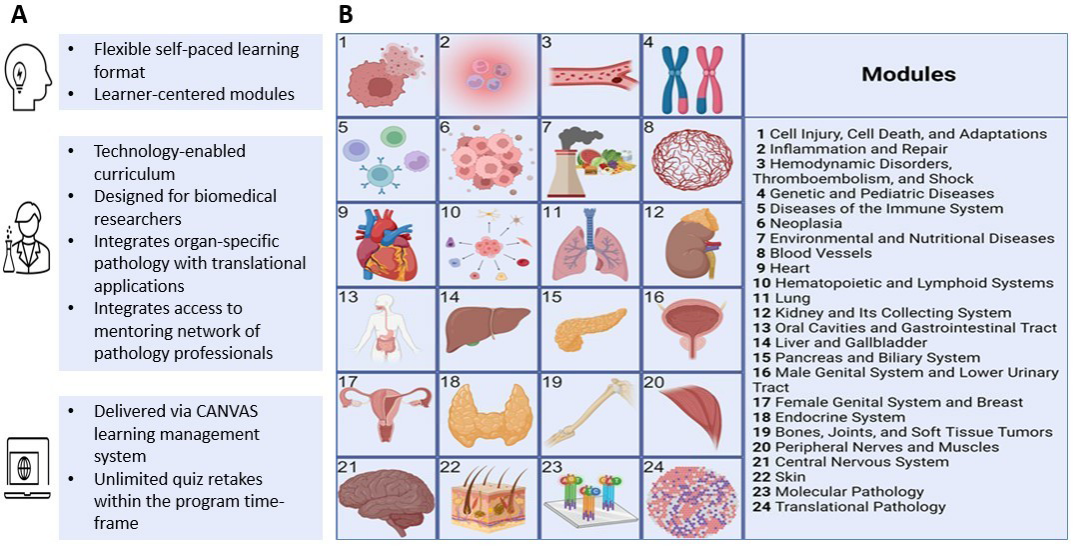
Fundamental Pathology for Basic Scientists: Course structure highlights (A) Curriculum overview (B). Shown in B are the 24 instructional modules for this course. Image created in BioRender (Sells, J., 2025 [13]), licensed by authors with Academic account.

Three key factors were taken into consideration when designing this course: (1) the broad target audience of basic scientists from various departments and academic career stages, with diverse training backgrounds and work roles, (2) the need for participants to complete this course alongside their full-time or regular work duties, and (3) course accessibility for participants from across the Texas Medical Center in Houston, Texas. The CANVAS Learning Management System was selected for the asynchronous delivery of course materials (Figure 1A), which consisted of lecture videos, presentation files, supplementary reading material (research publications, techniques, and method aids), and quizzes.

A total of 24 learning modules were organized, each focusing on topics of interconnecting physiopathology contents and clinicopathologic correlations with specific organ systems (Figure 1B). Traditional pathology topics were based on Robbins & Kumar Basic Pathology, Eleventh Edition, the international gold-standard text for pathological disease [14], and information from this book guided the selection of content for the majority of the modules (Modules 1‐22). While we endeavored to adhere to the sequencing of chapters as in this well-established teaching resource that is recognized in the field, each lecture, however, was tailored to our specific audience with varying levels of pathology knowledge, and the course also included 2 additional modules covering molecular pathology and translational pathology, not addressed in [14]. Module 23 on molecular pathology covered the definition of molecular pathology, molecular basis of disease, molecular pathology tools, diagnostic molecular pathology, and tumor molecular profiling. Module 24 on translational pathology covered introduction to translational pathology, -omics research, genomics, metabolomics, and other omics for target identification and validation, biomarkers in oncology, genetics and molecular biomarkers, and artificial intelligence to improve diagnostic and prognostic efficacy. The inclusion of dedicated modules in molecular pathology and translational pathology was not intended as an additive novelty in isolation, but as an explicit curricular bridge connecting traditional histopathology with contemporary research methodologies that are central to modern biomedical research but typically absent from foundational pathology training.

Furthermore, we incorporated Elsevier’s Evolve Resources for the Instructor-Led Course Robbins & Kumar Basic Pathology, Eleventh Edition [15]*,* which allowed us to tailor the content and learning experience to the unique needs of each participant, ensuring broad accessibility and learner engagement. Additionally, instructors enhanced each module by sharing relevant recent literature to reveal the incorporation and use of modern methodologies and instrumentation for pathology work.

This 24-module pilot course was offered over a span of 6 months. One module was released sequentially in CANVAS each week and included an estimated 1 hour of recorded video instruction on the topic, supplemented with relevant reading material from current literature. Participants were allowed to complete the module at their convenience, which provided considerable flexibility in accommodating their coursework into their ongoing work schedules and job responsibilities. Each module was created and taught by subject-matter experts from MD Anderson, and lecturers offered virtual office hours after every 4 modules to promote inquiry and interaction and follow-up on any additional learning resources the participants needed.

The professional roles of the instructors spanned the range from early-career assistant professors to associate professors, research scientists, and clinical pathology fellows to research postdoctoral fellows. All instructors were members of either the Pathology or Translational Molecular Pathology departments at MD Anderson and had received formal pathology training in the United States or internationally. Lecturers constructed a multiple-choice style quiz of 10 questions for each module, and learners were allowed multiple attempts to complete the quiz with a benchmark score of 80% for passing each module before they could progress to the next module. The 29 participants completed all 24 modules and were issued a certificate of completion at the end of the 6-month pilot course.

### Course Evaluation

Three surveys were issued to obtain pertinent insight. All surveys were conducted using the institutional Qualtrics management software. The first survey was a pre-entry questionnaire administered before the course, intended to collect background information from participants on departmental affiliation, prior training in basic pathology, and application of pathology reports or basic pathology in their current jobs or roles.

The second survey for evaluation was the exit survey, administered to each participant after completing all 24 course modules en route to gaining their course certificate. Aligned with a prominent, evidence-based practice for evaluating training programs, the exit survey was framed according to the Kirkpatrick Four-Level Evaluation Model [9,16] that has persisted as a gold-standard to the present day. The survey was subdivided to flow through key evaluation points on course structure, lecture delivery, module content, learning and knowledge application, and concluded with a free-response section for additional feedback. Each of the 4 Kirkpatrick levels (ie, 1: reaction, 2: learning, 3: behavior, and 4: results) were sequentially featured (Table 1).

**Table 1.** Topics surveyed for course evaluation and their alignment with the Kirkpatrick model of training program evaluation.

Relevance to Kirkpatrick level	Topics surveyed
1 Reaction	Duration of the course, learning objectives of each module and organization of course content, use of the virtual office hours, delivery format, course balance with work duties, format, and delivery of quizzes
2 Learning	Pathology-specific knowledge and skills gained, quizzes, confidence in applying their newly learned skills
3 Behavior	Relevance to their current jobs or roles and/or career-development goals, enhancement of current work duties
4 Results	Specialized pathology course interest, long-term value

The majority of the exit survey items were strategically geared toward Kirkpatrick Levels 1 and 2, as these are areas of immediate and high interest to the launch of a new training program. This evaluation was essential to fine-tune and tailor the modules in real-time for optimizing learning and instructional design tailored to meet our primary goal: to provide a baseline interface where nonpathologists whose everyday work eventually impacts pathology could gain pathology awareness. Level 3 (behavior) and Level 4 (results) metrics were captured in an additional survey, 1-year post-program completion. In addition to these participant perception-based metrics, checkpoint quizzes embedded at the end of each module served to objectively measure the knowledge gain - participants had to score 80% or better in order to proceed to the next module.

We also assessed our evaluation against a well-established interdisciplinary, problem-centered approach to using educational technology for learning design and instructional systems development [17], the Spector approach. Spector posed 5 levels of evaluation on the application of educational technology for instructional design when constructing a course or program in any discipline. Applying the Spector scale to the evaluation of this pilot course, our key metrics are shown in Table 2.

**Table 2.** Course evaluation criteria breakdown in alignment with the Spector scale for instructional program evaluation.

Spector level	Criteria for good learning and instruction	Supporting course statistic
1	Learning goals and objectives are met (formative and summative)	Course requirement ensured a minimum of 80% passing score per module [all 29 of 29 participants (100%) passed each of the 24 modules to receive a certificate of completion]; at least 26 out of 29 participants (90%) confirmed that the course modules had met their stated learning objectives
2	Learning setting is useful, and content aligned with stated objectives	At least 26 out of 29 participants (90%) confirmed that the course delivery format was favorable; at least 26 out of 29 participants (90%) confirmed the course duration was appropriate and that the distribution of instructional content in the models was favorable and aligned with the stated objectives
3	Overall course experience is satisfactory	27 out of 29 participants (94%) confirmed they would recommend this course to a colleague
4	Program is sustainable	Future direction to probe (long-term course iteration evaluation is necessary to determine course sustainability).
5	No harm is done	At least 15 out of 16 post-course survey respondents (94%) concurred that their self-knowledge in fundamental pathology has increased because of participating in this course. Future direction to probe long-term technological impact (planned 3-year post-course participant follow-up). A second cohort will allow for more in-depth Level 5 determinations to be made.

The final survey was conducted 1 year after program-completion, to assess long-term impact of the course on participants’ career and professional development, teaching abilities, and sustained confidence in basic pathology knowledge and skills.

### Ethical Considerations

This study was reviewed and approved by the Institutional Review Board (IRB) at the UT MD Anderson Cancer Center (OHRP IRB Registration Number: IRB00000121; Reference ID# 2024‐1381).

## Results

### Precourse Survey

Overall, 28 out of 29 participants (97%) who completed the course had previous biology training, and 21 out of the 29 participants (72%) had prior histology knowledge. Interestingly, while 23 out of 29 participants (79%) either used pathology reports or basic pathology concepts in daily work activities, only 24% of participants (7 out of 29) had previously received formal pathology training. Trainees (graduate students and postdoctoral fellows) only accounted for 9 out of 29 participants (31%), while 20 out of 29 participants (69%) were professionals in formal work capacities within the academic centers. A more detailed profile of the learners is provided in Table S1 in Multimedia Appendix 1.

### Course Exit Survey (Immediate or Near-term Training Impact)

**Figure 2.**
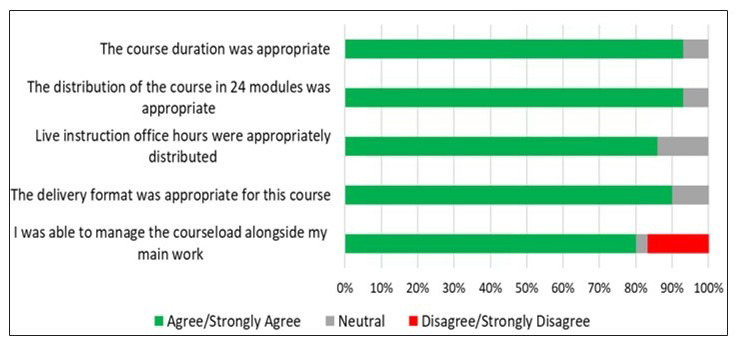
Fundamental Pathology for Basic Scientists: Data on participants’ perceptions of course structure. A Likert scale assessment of agreement with five statements evaluating the course duration, distribution of modules, delivery format, live instruction availability, and participant ability to manage course load (n=29).

All 29 course participants who completed the course completed the exit survey as well. A 5-point Likert scale was used to assess participants’ opinions on course structure, course content, and course lectures (Figure 2). For all questions, the scale anchors used were as follows: 1- strongly disagree, 2- disagree, 3- neutral, 4- agree, and 5- strongly agree. Since this was a small pilot study, the Likert anchors of agree or strongly agree were grouped together to enable ready visualization of positive responses, as they were the overwhelming majority of responses. Similarly, disagree or strongly disagree were grouped together to easily visualize negative responses since there were so few. At least 27 out of 29 participants (93%) concurred (agreed or strongly agreed) that the course duration and distribution of the course in 24 instructional modules was appropriate. When questioned about live instruction office hours, at least 25 out of 29 participants (86%) concurred that it was adequate and appropriately distributed. At least 26 out of 29 participants (90%) considered the course delivery format using lecture videos via CANVAS to be favorable, and at least 23 out of 29 participants (79%) reported being able to effectively manage the course requirements alongside their primary work.

**Figure 3.**
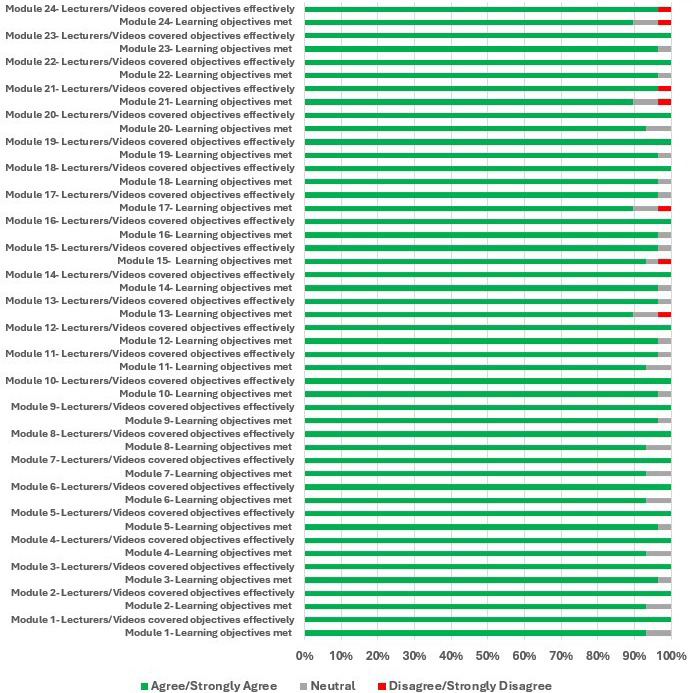
Fundamental Pathology for Basic Scientists: Data on participants’ perceptions of course effectiveness. A Likert scale assessment of agreement with two statements evaluating achievement of learning objectives and effectiveness of instruction for each individual course module. At least 89% of learners agreed or strongly agreed that the lecturers and videos covered the stated objectives effectively and that the learning objectives were met in each of the 24 modules (n=29).

The second section of the questionnaire surveyed participants’ opinions on course content. For each module, 2 statements were explored: “I feel that the learning modules met the stated learning objectives,” (*learning objectives met*) and “The lecturers of the course effectively covered the learning objectives in their lecture videos” (lecturers and videos covered objectives effectively). The detailed assessments are shown in Table S2 in Multimedia Appendix 1. Any of the negative Likert responses received were attributed to just one individual’s response of dissatisfaction within a given module. Overall, the feedback for both queries was strongly positive, with at least 89% of participants (at least 26 out of 29) concurring (agreeing or strongly agreeing) favorably with both statements for each of the 24 modules, underscoring the high quality of the instructional content, the superior teaching abilities of the instructors, and the strong alignment of the presented content with the stated learning objectives (Figure 3).

**Figure 4.**
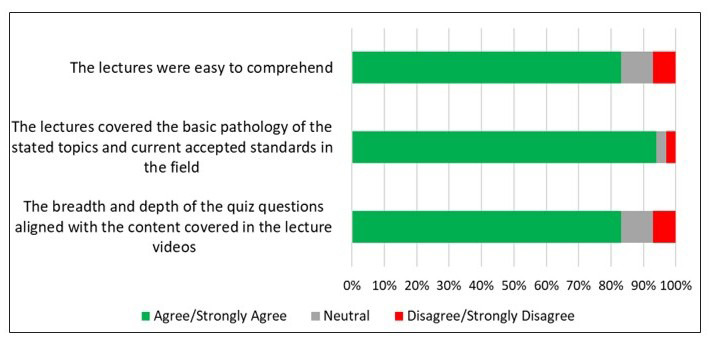
Fundamental Pathology for Basic Scientists: Data on participants’ perceptions of course lectures. A Likert scale assessment of agreement with three statements evaluating course comprehension, standards and adequacy, and alignment of course assessment quizzes with instructional content (n=29).

The third section of the exit survey focused on statements related to comprehension and standards of the course lectures, as well as the ability to address the quiz questions at module completion (Figure 4). In total, 83% of participants (24 out of 29) concurred (agreed or strongly agreed) that the lectures were easy to comprehend, and 27 out of 29 participants (94%) concurred that the lectures covered the basic pathology of the stated topics and matched currently accepted standards in the field. Eighty-three percent (24 out of 29) concurred (agreed or strongly agreed) that the breadth and depth of the quiz questions aligned with the content covered in the lecture videos.

**Figure 5.**
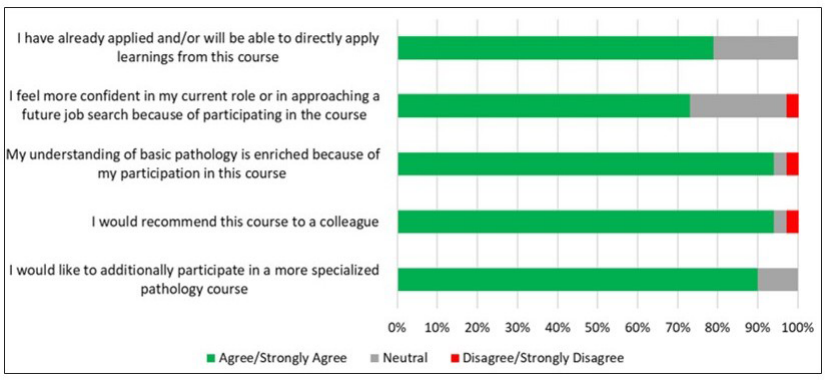
Fundamental Pathology for Basic Scientists: Data on participants’ perceptions of near-term impact of course on professional development and career support. A Likert scale assessment of agreement with five statements evaluating course applicability to current career stage, confidence in job search, knowledge and skill development, and sustained interest in returning for an advanced course in pathology (n=29).

The final section of the exit survey assessed the overall impact of the course on participants’ careers and the potential application of what they learned in their current roles (Figure 5). Seventy-nine percent of participants (23 out of 29) concurred (agreed or strongly agreed) that they have either already applied or will be able to directly apply the content learned from this course to their current roles. Twenty-one out of 29 participants (72%) concurred (agreed or strongly agreed) that participation in this course made them feel more confident either in their current role or in approaching future job searches along their career trajectory. Twenty-seven out of 29 participants (94%) concurred (agreed or strongly agreed) that participation in the course directly enhanced their basic pathology knowledge. Further, 27 out of 29 participants (94%) also concurred that they would recommend this course to a colleague. Notably, 26 out of 29 participants (90%) concurred that they would like to return to participate in a more specialized pathology course (advanced topics in pathology, such as neuropathology, advanced tissue imaging and analysis techniques, animal disease model pathology, histology staining and processing techniques, histology interpretation, hematopathology, and cytopathology were suggested as content for future specialized course options), highlighting the positive impact of the pilot training course.

We also received free response feedback from 66% of participants (19 out of 29), regarding potential opportunities for course improvements in future iterations. Suggestions included the provision of additional relevant journal articles, the creation of a discussion board for learners to engage in conversations around pathology-related discussion prompts, providing more detailed explanations for images, varying the types of questions in quizzes, and collaborative activities among learners.

### Postcourse Survey (Long-term Impact on Career and Professional Development)

A 5-point Likert scale was applied, with questions using the following scale anchors: 1- strongly disagree, 2- disagree, 3- neutral, 4- agree, and 5- strongly agree. Similar to the end-of-course survey, considering this is a small pilot study, the agree or strongly agree responses were combined into a single metric for ease of visualization of positive versus negative responses.The disagree or strongly disagree responses were similarly grouped together. Of the 29 participants who graduated from the pilot course, 55% (16 out of 29) completed postcourse survey responses a year after program graduation. This attrition in long-term follow-up is acknowledged; however, given that extended postprogram follow-up is a common hurdle in the field [18-20], this is an expected limitation. Nevertheless, obtaining feedback from over half of the program graduates was very valuable and will inform the design of the upcoming second course iteration.

**Figure 6.**
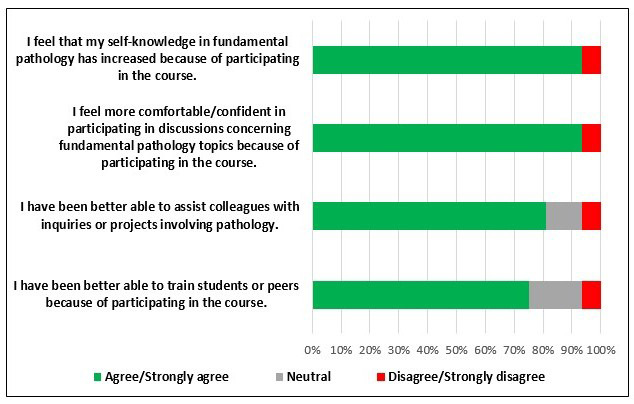
Fundamental Pathology for Basic Scientists: Data on longer-term (One-year post-program completion) impact of course on participants’ pathology knowledge and teaching skills. A Likert scale assessment of agreement with four statements evaluating longer-term use of learned pathology knowledge and skills (n=16).

Overall, 50% of the respondents (8 out of 16) had had a job change within the past year, and 81% (13 out of 16 respondents) concurred that their current jobs or roles involved pathology (data not shown). Moreover, 15 out of 16 respondents (94%) concurred (agreed or strongly agreed) that they felt more confident in participating in discussions concerning fundamental pathology topics at work (Figure 6). Fifteen out of 16 respondents (94%) also concurred that their knowledge in fundamental pathology has increased because of participating in this course (Figure 6). Feedback in the form of free responses indicated that the course helped learners identify and differentiate the distinct cell types within tissues, better understand mechanisms of resistance, better understand research results, quality assurance, better understand microscopic histology techniques, immunohistochemistry data, disease processes at a cellular level, as well as better understand normal tissue structure that has helped in recognizing tissue abnormalities. Learners also stated that this course made them more comfortable with pathology, made it easier to connect scientific concepts to real-world applications, and helped open career opportunities.

Extending to knowledge applications on the job, 13 out of 16 respondents (81%) concurred that they were better able to assist colleagues with inquiries or projects involving pathology and 12 out of 16 respondents (75%) agreed or strongly agreed that they were even better able to train students or peers because of participating in this course (Figure 6). Free response feedback also illuminated more avenues for course improvement, suggesting the need for better clarity in image descriptions within the video lectures.

## Discussion

### Principal Findings

The lack of accessible, structured learning opportunities in fundamental pathology creates a significant knowledge deficit that can limit career advancement for basic and translational scientists within the health care research enterprise. Furthermore, the rapid pace of advancements in biomedical research emphasizes the need for continuing education in pathology to ensure that professionals stay abreast of the latest developments. It is also imperative for such workplace training initiatives to embrace and integrate digital tools and virtual learning to solve access issues in the setting of the post-COVID-19 pandemic [21]. As the first iteration of the Fundamental Pathology for Basic Scientists course, the focus of this pilot study was on determining the value of the instructional design and program delivery to fill this training gap. Main findings yielded evaluative data on categories of course structure, course content, course effectiveness, course lectures, near-term impact of the course on professional development, and long-term impact on pathology knowledge applications. Line items within each category were crafted to obtain critical appraisal on overall course design and personal user aspects such as confidence in using the knowledge gained, as well as the actual application of learned skills on the job. Participant responses reflected strong endorsement of this course in terms of user value and use.

Notably, the course was developed using local institutional resources. It was able to draw participants from 16 different academic departments, representing 10 distinct job roles within the biomedical research enterprise at Texas Medical Center, ranging from early-career scientists to more established professionals. The majority of participants (22 out of 29 participants, 76%) lacked formal training in pathology. (Table S1 in Multimedia Appendix 1). The wide-ranging diversity of enrollees underscores the broad relevance of pathology knowledge across various career stages and disciplines. It also reflects the interdisciplinary nature of modern biomedical research, where a shared understanding of pathology can facilitate more effective collaboration and innovation. The positive feedback from participants, along with the heterogeneous mixture of departments and career stages represented, highlights the unmet demand for pathology education tailored to the needs of working professionals within the workplace setting. This demand suggests that similar courses could be highly beneficial if made more widely available, particularly in online, self-paced formats that accommodate the busy schedules of various professionals. Our study confirms and extends previous reports in the literature [22,23], showing that readily-accessible (such as locally-curated) educational resources would help close the knowledge gap in pathology and empower a broad audience of scientists and health care professionals to apply this critical education in their respective domains, and also facilitate advanced and interdisciplinary career opportunities within broader biomedical research disciplines (eg, teaching) (Figures 5 and 6).

The course’s comprehensive curriculum, covering an extensive range of basic pathology topics, was one of its key strengths, with the benefits continuing to grow for participants even after a year of course completion (Figures 3 and 6). The observation that at least 26 out of 29 participants (89%) uniformly concurred that each of the 24 modules had met its stated learning objectives confirms the appropriateness of the instructional content. The evaluation method for course content primarily involved quizzes integrated into each learning module. These quizzes served as both an assessment tool and a reinforcement strategy, ensuring that participants engaged with and retained key concepts. This approach aligns with educational research that supports the use of frequent, low-stakes quizzes to enhance knowledge retention and promote active learning [24,25]. Participants responded positively to this form of assessment, appreciating the immediate feedback and the alignment of quizzes with the course content.

The observation that 93% (14 out of 16) of postcourse survey respondents felt this course significantly enhanced their knowledge and skills and further made them more confident in approaching projects involving pathology or even engaging in pathology-related discussion (Figure 6) demonstrates long-term impact of this course, and also its practical value for application across a wide spectrum of biomedical branches. Further, this highlights the course’s ability to meet the diverse needs of a varied audience and reinforces its importance as a vital and readily-accessible resource for professionals seeking to deepen their understanding of pathology in the context of their work. One limitation of our evaluation is that we were able to receive the long-term survey responses from 55% (16 out of 29) of program graduates. While this attrition in contacting program alumni is indeed well described in the literature and is not uncommon [18-20], we anticipate being better positioned to evaluate and mitigate potential response bias, as the program expands with increased participant numbers in future iterations.

### Opportunities for Program Enhancement in Future Iterations

The course, delivered over 6 months with 24 weekly modules, was designed to be flexible through an asynchronous format, allowing participants to integrate learning with their ongoing professional duties. While the course was effective in meeting its educational objectives, feedback indicated that managing the course workload alongside professional responsibilities remained a significant challenge for at least 6 out of the 29 participants (20%), suggesting that the course structure could benefit from further refinement. Several strategies could improve the course experience in future iterations: Extending the overall course timeline to 8 or 10 months would provide participants with more time to complete each module, making it easier to balance their educational pursuits with their professional duties. Modifying the module release schedule, by releasing modules biweekly or in clusters, instead of weekly, would offer participants greater time and flexibility in managing their schedules. Additionally, incorporating optional review periods or breaks between certain modules could serve as catch-up periods, reducing the pressure of keeping up with a weekly release schedule. Providing time management resources or strategies, such as workshops or tips on integrating learning with daily routines, would further help participants balance the course with their work. Regular feedback from participants could also be used to make real-time adjustments to the schedule or workload, ensuring the course remains manageable for all participants.

Some participants suggested incorporating more diverse question types, such as case-based questions or scenario analysis, to better simulate real-world applications of pathology knowledge. Future iterations of the course will expand the quiz format to include a broader range of question types and consider adaptive quizzes that adjust in difficulty based on participants’ responses.

Further, while the course’s overall curriculum was well received, some participants expressed a desire for additional specialized topics, particularly in emerging areas of translational pathology. This could include advanced diagnostic techniques or deeper explorations of subspecialties like neuropathology or hematopathology. We view this as feasible because our course instructors are active professionals within the Texas Medical Center community who participate in continuing pathology education, pathology conferences, and/or meetings to keep up-to-date with the rapidly evolving landscape in pathology methods and research. There were also suggestions for more interactive elements, such as case-study discussions or group projects, to enhance engagement and deepen exploration of the material. Incorporating occasional synchronous sessions, despite the overall asynchronous format, could also provide a more dynamic learning experience. Finally, although virtual group office hours were offered, additional virtual availability with instructors on a one-on-one basis at the request of the participants seemed necessary to address inquiries or other ongoing course needs. Future iterations will seek to incorporate these suggestions and improvements into the curriculum.

### Conclusions

The Fundamental Pathology for Basic Scientists course represents a local institution-based immersive learning tool to effectively address a critical educational gap in pathology for basic- and translational scientists. Emphasis was placed on learners acquiring a better sense of self-learning on pathology language and concepts as opposed to objectively measured cognitive competencies alone. Because participant makeup was explicitly nonpathologists, the building of their practical self-perceptions is a critical first step. While the pilot course was successful in achieving its training goals, multiple opportunities for course enhancement were identified from participant feedback. Future iterations of the course will consider extending the timeline, offering more specialized content, enhancing the image descriptions, and increasing interactive elements to enhance the learning experience. Regularly soliciting participant and instructor feedback will be crucial in continuously improving the course and ensuring it remains a valuable resource for the next generation of basic scientists and health care professionals.

## Supplementary material

10.2196/84903 Multimedia Appendix 1 Supplementary tables detailing the pre-entry survey outcomes and participants' evaluation of course content.

## Abbreviations

UT: University of Texas
