# Supplementary material for: Enhancing Pathology Knowledge for Biomedical Scientists: Pilot Cohort Study of a Comprehensive Self-Paced Tutorial for Bridging Fundamental Concepts With Translational Applications
Source: JMIR Med Educ. 2026 May 21;12:e84903. doi: 10.2196/84903 (PMC13193296; doi:10.2196/84903)
Supplement: Multimedia Appendix 1 [file mededu-v12-e84903-s001.docx]

**Enhancing Pathology Knowledge for Biomedical Scientists: A Comprehensive Self-Paced Online Curriculum for Bridging Fundamental Concepts and Translational Applications**

Sells et al.

**Table S1.** *Fundamental Pathology for Basic Scientists* pilot course: Pre-entry survey outcomes with participant information, academic career stage and prior training background

**Table S2.** *Fundamental Pathology for Basic Scientists* pilot course: Course content evaluation *per* instructional module
